# Immunoprofiling of Rice Root Cortex Reveals Two Cortical Subdomains

**DOI:** 10.3389/fpls.2015.01139

**Published:** 2016-01-07

**Authors:** Sophia Henry, Fanchon Divol, Mathilde Bettembourg, Charlotte Bureau, Emmanuel Guiderdoni, Christophe Périn, Anne Diévart

**Affiliations:** CIRAD, UMR AGAPMontpellier, France

**Keywords:** rice root, cortex, markers, antibodies, lateral roots, tissue identity, confocal microscopy, confocal imaging

## Abstract

The formation and differentiation of aerenchyma, i.e., air-containing cavities that are critical for flooding tolerance, take place exclusively in the cortex. The understanding of development and differentiation of the cortex is thus an important issue; however, studies on this tissue are limited, partly because of the lack of available molecular tools. We screened a commercially available library of cell wall antibodies to identify markers of cortical tissue in rice roots. Out of the 174 antibodies screened, eight were cortex-specific. Our analysis revealed that two types of cortical tissues are present in rice root seedlings. We named these cell layers “inner” and “outer” based on their location relative to the stele. We then used the antibodies to clarify cell identity in lateral roots. Without these markers, previous studies could not distinguish between the cortex and sclerenchyma in small lateral roots. By immunostaining lateral root sections, we showed that the internal ground tissue in small lateral roots has outer cortical identity.

## Introduction

Rice has a complex root architecture with a mix of embryonic and post-embryonic roots. The radicle emerges first during germination, followed soon thereafter by embryonic coronary roots (Rebouillat et al., 2009; Coudert et al., 2010). The first adventitious post-embryonic roots resulting from shoot apical meristem activity appear a few days after seedling germination. Adventitious roots are also called coronary or crown roots and are produced throughout the life of a plant.

**Figure 1:**
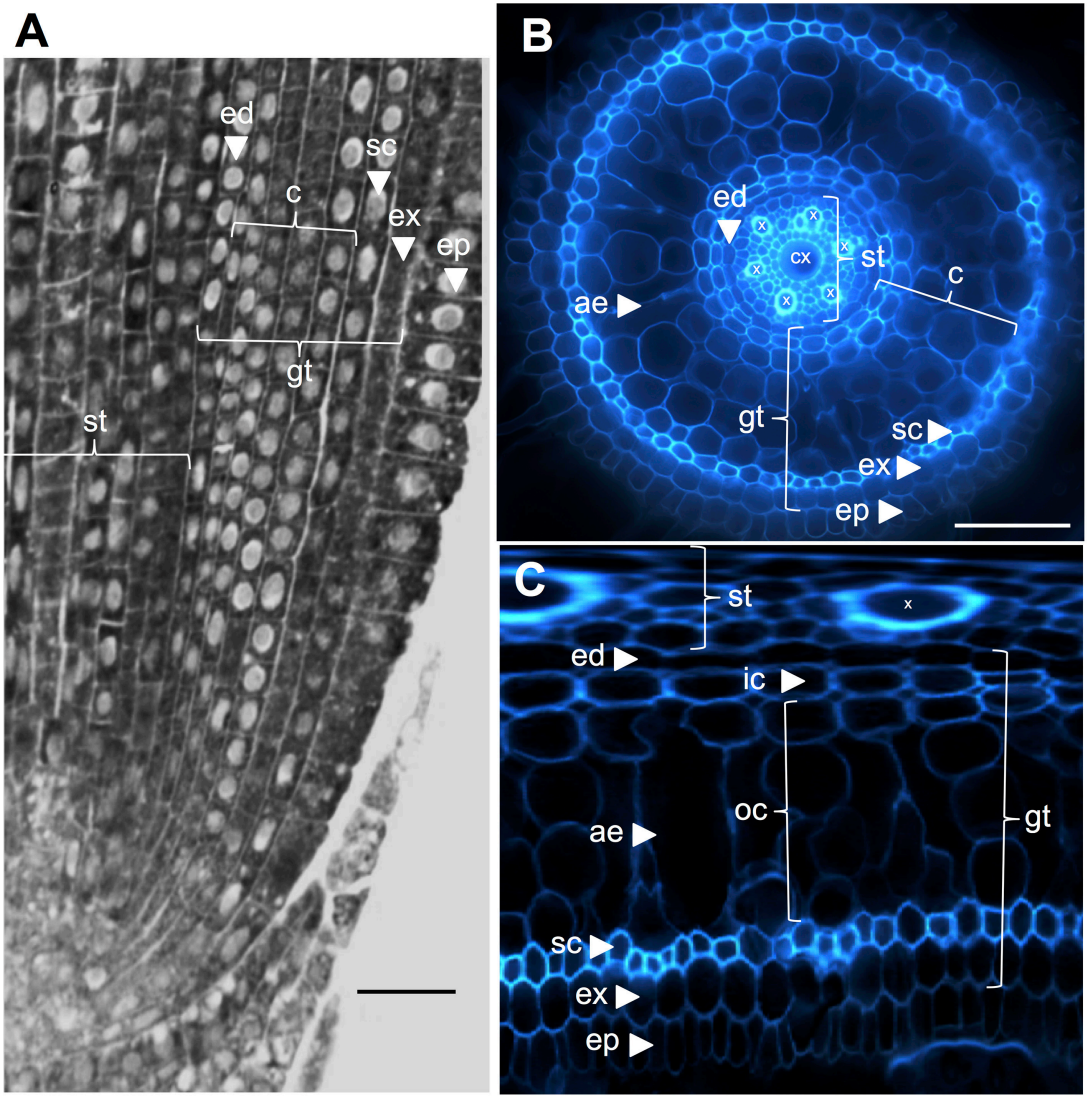
**Anatomy of a 6-day-old Nipponbare radicle. (A)** Longitudinal section of a radicle fixed and paraplast embedded. Bar = 100 μm. **(B)** Transverse section of a fresh radicle observed under UV light. Bar = 50 μm. **(C)** Polar transformed view of B. Layers of tissues are labeled as follows: stele (st), ground tissue (gt) composed of several cell layers: one layer of endodermis (ed), several layers of cortex (c), one layer of sclerenchyma (sc), and one layer of exodermis (ex). The endodermal (ed) and exodermal (ex) layers are easily identified by the absence/reduction of fluorescence in the cell wall centers of these cells. The radicle is protected by one layer of epidermis (ep). Two types of cortical layers can be identified: inner cortex (ic), which is next to the endodermis (ed), and outer cortex (oc), which will eventually form aerenchyma (ae). In the stele, note the presence of a large central metaxylem (cx) surrounded by 6 metaxylem (x) vessels.

The internal rice root anatomy is well-described, particularly at the seedling stages (Rebouillat et al., 2009). Several concentric tissues (from the periphery to the stele) are located between the epidermis and the vascular tissues (Figure 1). These tissues, collectively referred to as “ground tissue,” are sometimes also referred to as the “cortex” (Lux et al., 2004). Here, we retain the generic term, i.e., ground tissue, to describe the exodermal, sclerenchyma, cortical, and endodermal tissues. We refer to the “cortex” as the tissue located between the endodermis and the sclerenchyma.

Ground tissue is composed of several layers of tissues that have the same parenchymatous origin but different specific characteristics that play various and important structural and functional roles in roots. For example, the exodermis and endodermis are apoplastic barriers that control the radial transport of water and nutrients from the soil to the photosynthetic organs, a feature mostly provided by suberin lamellae deposition and secondary cell wall differentiation (Gregory, 2006). Sclerenchyma are lignified cells that represent a critical barrier to some metals and possibly play a mechanical support role (Huang et al., 2012). In contrast to these three monolayer tissues, the cortical tissue in the rice radicle is composed of four or five layers. This tissue, also named the mid-cortex or mesodermis, is characterized by the schizogenous formation of spaces, leading to the formation of aerenchyma in rice (Justin and Armstrong, 1991; Kawai et al., 1998). Aerenchyma plays a major role in tolerance to flooding conditions and ensures continuous oxygen flow from the shoot to the root, allowing root growth under anoxic conditions. The cortex has an important but variable function in root physiology of numerous species and is involved in storage, flooding tolerance, or symbiotic interactions (Lux et al., 2004).

The radicle and crown roots produce three types of lateral roots: small lateral roots (SLRs) and two types of large lateral roots [Thin (T-LLRs) and Large (L-LLRs)] (Kawata and Shibayama, 1965; Kono et al., 1972; Rebouillat et al., 2009). This division is based on (i) root diameter: SLRs are the smallest (50–60 μm) and L-LLRs are the largest (120–150 μm), (ii) ramifications, which are only observed on LLRs, (iii) the presence of a central metaxylem vessel only in LLRs, and (iv) the presence of a sclerenchyma layer only in L-LLRs (Kono et al., 1972; Kawata et al., 1977; Rebouillat et al., 2009). Despite this classification, the identity of internal root tissues in lateral roots, particularly SLRs, remains uncertain. Indeed, Rebouillat et al. (2009) and Kawata et al. (1977) both claimed that SLR ground tissue was comprised of the endodermis, sclerenchyma and exodermis (from the inside toward the outside), while in Kono et al. (1972), the inner-cortex sclerenchymatous cell layer was described as absent in SLRs and T-LLRs.

To solve this dilemma, and to study the development and function of particular tissues in general, the use of identity markers is required. Most of the tissue markers used until now are morphological markers or histochemical stains, which have provided a useful way to characterize cell identity. Berberine reveals the presence of suberin in cell walls, which is found almost exclusively in the Casparian strips that surround each individual endodermis cell and is thus a good indirect marker of endodermal identity (Brundett et al., 1988). In *A. thaliana*, tissue-specific markers were also developed using tissue-specific promoters fused to reporter genes (GUS and/or GFP) in genetically modified plants. For instance, the *SCARECROW* promoter is used as an endodermal identity marker (Sugimoto et al., 2010). QC25 and QC46 enhancer trap GUS lines (Sabatini et al., 2003) and QHB in rice (Kamiya et al., 2003) were also identified as quiescent center (QC) markers based on their QC-specific expression. Despite interest in these markers, only a few are available and almost exclusively in *A. thaliana*. They also have drawbacks. For instance, the suitability of using the *SCARECROW* (*SCR*) promoter as an “endodermis” marker is debatable when specific questions on SCR and SHORT ROOT (SHR) roles in ground tissue differentiation are addressed. Indeed, the *SCR* and *SHR* genes are involved *per se* in endodermis/cortex formation (Wu and Gallagher, 2014). In *A. thaliana*, the only available root cortex markers are the *Co2* and *Co3* promoters (Heidstra et al., 2004; Ten Hove et al., 2010). In rice, these cortex markers are not yet used because there are no clear orthologs for the *Co2* and *Co3* genes. Only indirect morphological markers have been used to date in rice, such as aerenchyma formation for cortical identity (Rebouillat et al., 2009).

Another class of markers is commercially available antibodies directed against plant cell walls (http://www.plantprobes.net/index.php). These markers have been used scarcely in the past, but their efficiency has been demonstrated. For instance, the CCRC-M2 and JIM13 cell wall antibodies were used in *A. thaliana* to show that the single layer of internal tissue present in the *shr* mutant had cortical identity while the ground tissue layer of the *scr* mutant exhibited multiple identities (Di Laurenzio et al., 1996; Helariutta et al., 2000). These markers have many advantages: their simplicity and the possibility of combining secondary antibodies coupled to different fluorochromes to limit overlapping with auto-fluorescence, they do not require genetic transformation and they can be used to complement other classes of markers.

In this paper, we first describe a simple medium-throughput protocol for immunolabeling fresh vibratome tissue sections. Using this protocol, we screened radial sections of rice radicles with a large library of 174 cell wall antibodies. Among these, we identified eight cortex-specific markers and demonstrated that rice possesses two types of cortical tissues with distinct identity. We called these layers the inner and outer cortex based on their respective anatomical position in the root sections. To demonstrate the usefulness of these cortex-specific markers, we clarified the ground tissue identities of lateral roots using three of these antibodies. In SLRs in particular, we show that the internal tissue layer has an outer cortical identity. Our protocol is generic enough to be used for the development of tissue markers in any species without the need to generate transgenic plants. This protocol can be easily adapted with fresh shoot, leaf, root, or any tissues in other plants and represents a simple and easy way to identify tissue-specific markers. Moreover, these markers can be used in conjunction with other molecular markers.

## Results

### Immunoprofiling of cell wall antibodies in rice root radial sections reveals specific antibodies for cortex layers

**Figure 2:**
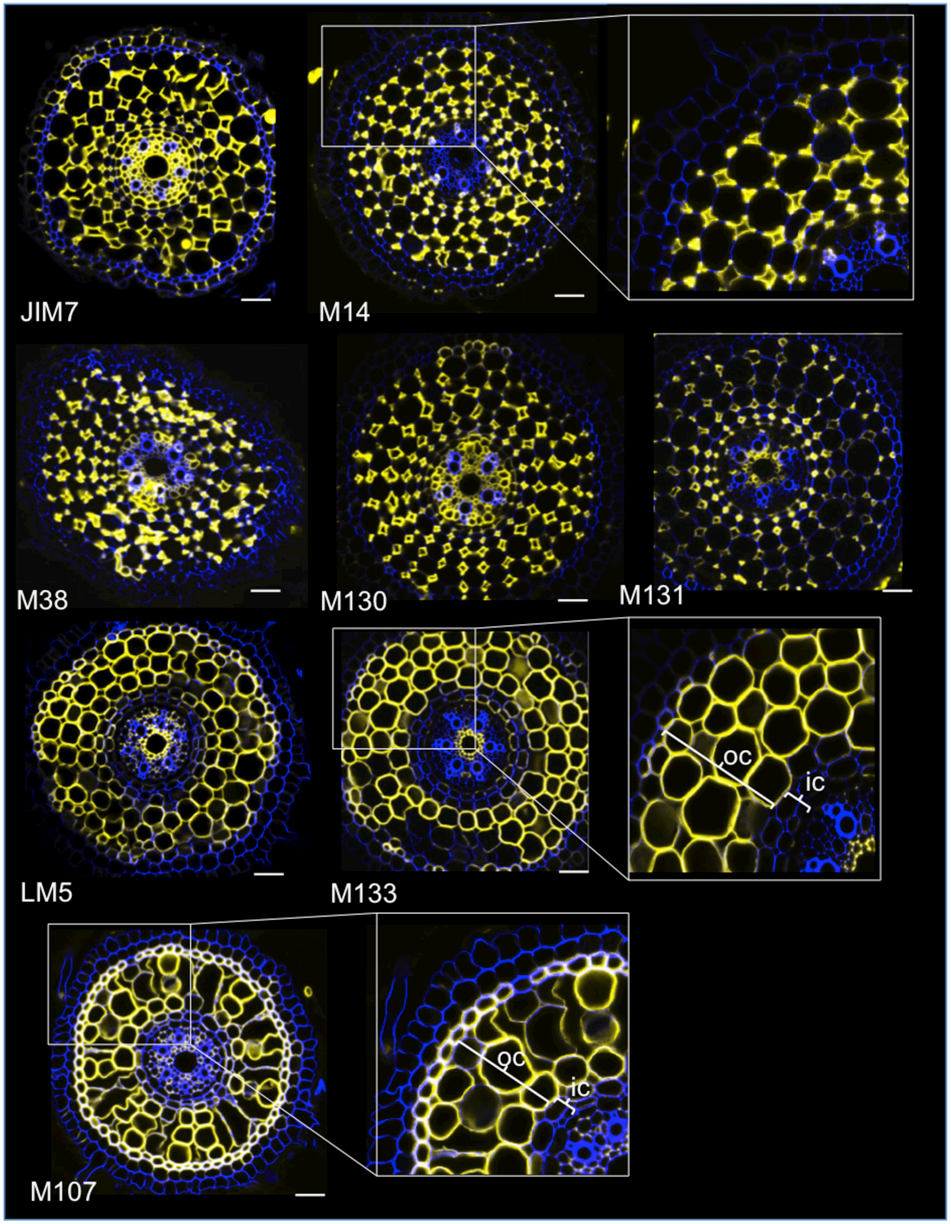
**Identification of eight specific cortex antibodies (JIM7, M14, M38, M130, M131, LM5, M133, and M107) among the 174 screened from the monoclonal cell wall antibody library**. For each antibody, merged images of transverse sections were obtained under a confocal microscope after immunohistological labeling: in yellow, antibody pattern at 561 nm, and in blue, cell wall autofluorescence under UV light. Five antibodies (JIM7, M14, M38, M130, and M131) show a similar profile within all cortical cell layers stained (diamond shapes). Three antibodies (LM5, M133, and M107) homogeneously labeled cortical cell walls. Note that for these antibodies, inner cortical (ic) layers are not stained compared with outer cortical (oc) layers. Insets are close-ups of the corresponding images. Bars = 20 μm.

With the aim of identifying antibody-based markers for cortical cells, we first established a protocol for medium throughput immunolabeling of radial sections of fresh rice roots (see Materials and Methods and Supplementary Figure 1 for details). Then, we performed a screen with monoclonal antibodies raised against cell wall components (from the Complex Carbohydrate Research Center (CarboSource Services, Athens Georgia, USA) and Paul Knox Cell Wall lab (university of Leeds, UK), see Materials and Methods for details)) to identify cortex markers. From the 174 antibodies tested, only 12 were retained as putatively cortex specific. We tested their repeatability by performing two additional experiments and concluded that eight antibodies displayed similar and robust profiles among the three replicates (Figure 2). Five antibodies (JIM7, M14, M38, M130, and M131) provided the same immunolabeling pattern in the cortex. Only non-adjacent cell walls were labeled, resulting in a singular “diamond shape” labeling of all cortical cells. For the three other antibodies (LM5, M133, and M107), the labeling was detected in the cortex and sometimes the sclerenchyma cells, particularly with the LM5 and M107 antibodies. The signal was homogeneous on the cortex cell wall, but was much weaker in the inner cortex layers (the layer of cortex adjacent to the endodermal layer) than in the outer cortex layers. Thus, all of these antibodies with profiles that are complementary to cortical tissues are specific markers that can be used to analyze cortical cell identity. Moreover, the two different cortical labels, defining two subcortical areas, suggest that the outer and inner layers have a distinct molecular identity that was revealed by these antibodies.

Some specific features of these two subcortical layers were highlighted when the “polar transformer” plugin of the ImageJ software (http://imagej.nih.gov/ij/) was used to view the images of the root sections (Figures 1B,C; Schneider et al., 2012; Lartaud et al., 2014). The inner cells were characterized by a thick cell wall compared with the outer cells, which exhibited various air-containing cavities resulting from cell wall fusion. The inner cortical cells also exhibited a flattened shape resembling endodermal cells, in contrast to the outer cells, which were rounder. In this transformed picture, the radial cell wall that is wall running from the inner side of the cell to the outer side, autofluorescence of the endodermal and exodermal cells are not visible, presumably due to the presence of Casparian strips. Similarly, but to a lesser extent, small holes exempt of autofluorescence were also visible on the radial cell walls of inner cortical cells.

### All lateral root types possess only outer cortical tissue

**Figure 3:**
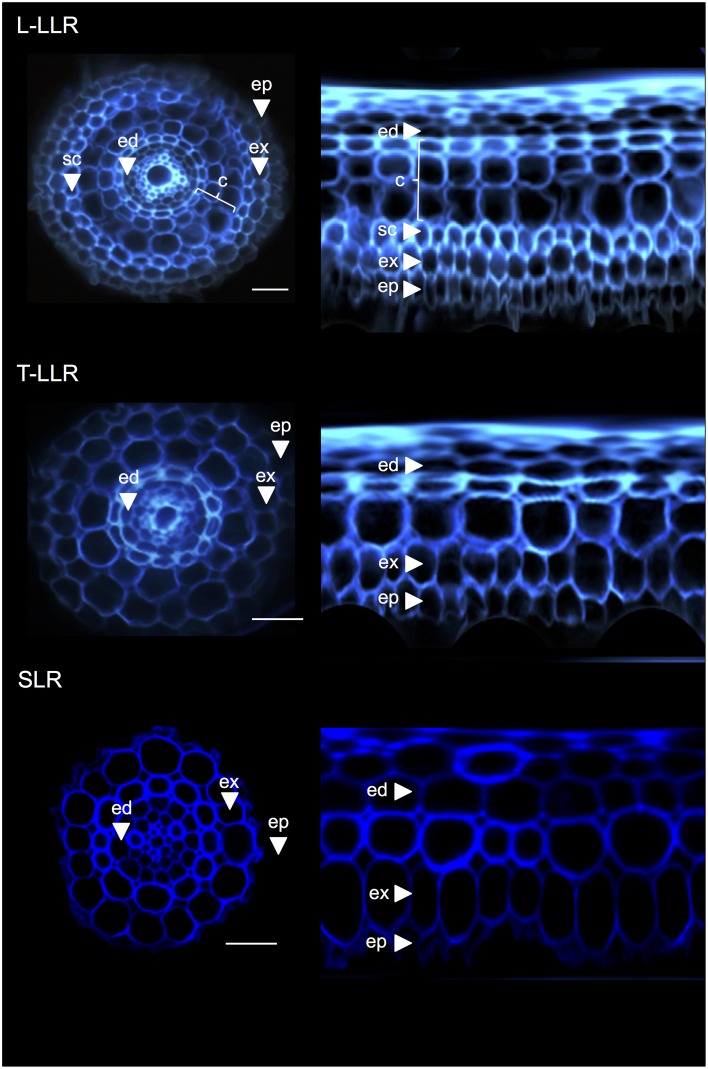
**Tissue composition of the Large Lateral Root types (Large-LLR and Thin-LLR) and Small Lateral Root (SLR)**. Seedlings were grown under hydroponic conditions for 2 weeks. Cell wall autofluorescence of transverse sections and polar transformed view under UV light. Tissues are labeled as follows: epidermis (ep), exodermis (ex), cortex (c), endodermis (ed). Note that the epidermis (ep) was often fragmented during the course of the experiment. Bars = 20 μm.

To demonstrate the usefulness of the cortex markers and to characterize cell identities, we labeled the three rice lateral root types (L-LLR, T-LLR and SLR) with three of these antibodies (M107, M133, and M14). For this experiment, because L-LLRs do not often develop on Petri dishes, lateral roots were collected from rice seedlings grown under hydroponic conditions for 2 weeks. We first analyzed the ground tissue organization of each type of lateral root by observing the autofluorescence of the cell walls in the transverse and polar views of the root sections (Figure 3). This first analysis revealed slightly distinct internal radial anatomy for the three types of lateral roots. Similar to the radicle sections viewed under UV light, the endodermal, and exodermal tissues can be identified by the fluorescence extinction in the center of the radial cell walls. These two tissues were present in all lateral roots. Between the endodermis and exodermis, several tissue layers were present in the L-LLRs and T-LLRs while the SLRs possessed only a single (unidentified) cell layer. The anatomy of the L-LLRs looked like seminal roots and external to the endodermis, several layers of cortical tissue were easily identified when aerenchyma tissue was formed. In addition, a sclerenchyma layer formed of closely packed cells with wide cell walls was also observed. Depending on the experiment, this cell layer was not always completely developed. In the T-LLR sections, the external layer resembled cortical tissue and the internal layer resembled the single (unidentified) SLR layer.

**Figure 4:**
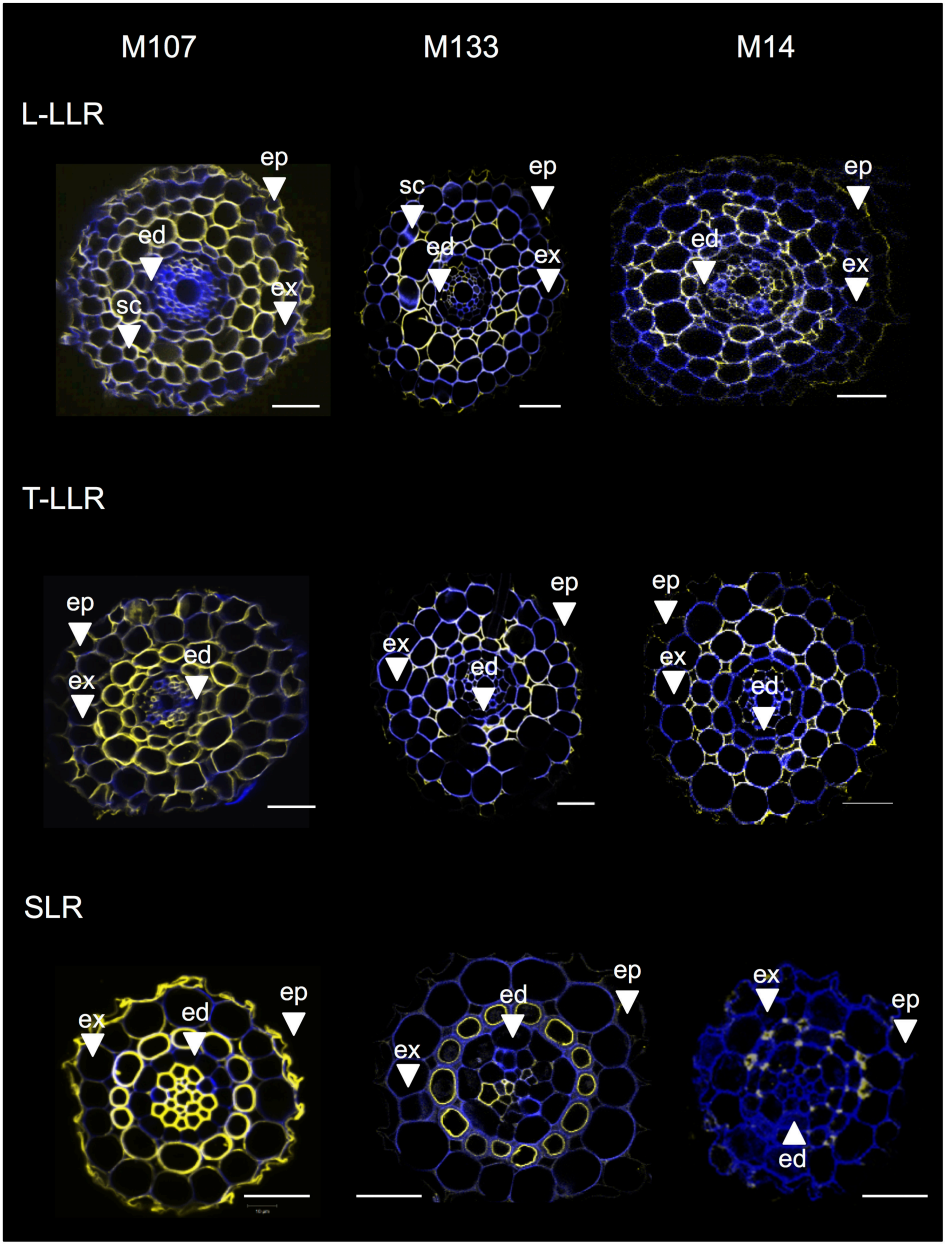
**Merged images of the Large Lateral Root types (Large-LLR and Thin-LLR) and Small Lateral Root (SLR) sections immunolabeled with M107, M133, and M14**. Seedlings were grown under hydroponic conditions for 2 weeks. In yellow, antibody labeling at 561 nm, and in blue, autofluorescence under UV light. Tissues are labeled as follows: epidermis (ep), exodermis (ex), cortex (c), endodermis (ed). Note that the epidermis (ep) was often fragmented during the course of the experiment. Bars = 20 μm.

To identify the cell layers present in the SLR and T-LLR ground tissues and to confirm the cortical identity in the L-LLRs, we immunolabeled lateral root sections with three cortex-specific antibodies (M107, M133 and M14; Figure 4). The M107 and M133 antibodies labeled all cell layers located between the endodermis and exodermis in all lateral roots. Based on radicle staining for these antibodies, this observation indicated that these cell layers could have sclerenchyma or cortical identity. However, the fact that M14 also immunolabeled these layers, particularly in the SLR sections, clearly demonstrates that these layers had outer cortical identity.

**Figure 5:**
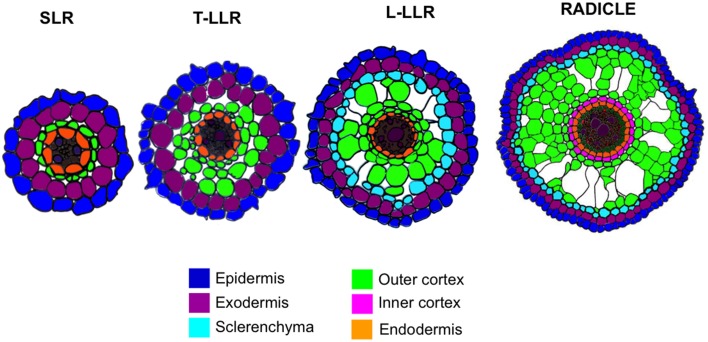
**Schematic representation of the lateral root and radicle tissues**.

Variations in the number of outer cortical cell layers (one in the SLRs, two in the T-LLRs, three in the L-LLRs and four to five in the radicle) play a major role in rice root diameters (Figure 5).

## Conclusions

Different terms have already been used to qualify subcortical areas in plants. For example, the presence of an “inner” lignified cortex layer, comparable to the one studied here, has already been described in wild rice (Yang et al., 2014). In maize, the two cortical domains are defined differently (Baluška et al., 1993). The “inner” domain forms aerenchyma, corresponding to the outer cortex here, and the “middle-outer” cortex is a lignified support tissue, which could be sclerenchyma. In *Arabidopsis*, a “middle cortex” cell layer, located between the cortical monolayer and the endodermis, has also been described (Baum et al., 2002; Paquette and Benfey, 2005). Recent results suggest that this cortex layer, whose formation is regulated by GA, oxidative stress, and SHR and SCR among others, plays a role in abiotic tolerance (Paquette and Benfey, 2005; Cui and Benfey, 2009; Pauluzzi et al., 2012; Wu and Gallagher, 2014). Interestingly, this layer is also described as a helix-like layer with a typical shape (Baum et al., 2002). Could this *Arabidopsis* “middle cortex” layer be functionally and structurally similar to the inner cortex layer of the rice radicle? In longitudinal and transverse sections of rice, the inner cortical layer also usually forms a helix, suggesting these two tissue layers could be comparable. In *Arabidopsis*, the “middle cortex” layer is derived from an endodermis periclinal division, which appears away from the QC between 7 and 14 days after germination (Paquette and Benfey, 2005). New tools will be necessary to verify these important parameters in rice. For example, no mutant affecting the timing of formation of this layer has been described up to now. Moreover, technical advances in imaging the rice QC are also required. Nevertheless, our cortex-specific markers offer a new tool to compare the function and molecular network involved in inner cortex (rice) vs. “middle cortex” (*Arabidopsis*) formation. Work is ongoing in our laboratory to identify the molecular determinants of inner and outer cortex formation, with SCR and SHR rice orthologs as first candidates for the regulation of cortex formation in rice roots (Pauluzzi et al., 2012).

## Materials and methods

### Plant material

#### Six-day-old seedlings

Seeds of the *Oryza sativa* L. ssp. *japonica* Nipponbare cultivar were grown vertically in sterile Petri dishes (Corning, 431301; 20 × 20 cm) under controlled conditions (day/night rhythm: 12/12 h, 28/25°C, light intensity: 500 μE m-2 s-1). First, the seeds were surface-sterilized by rinsing in 70% ethanol for ~1 min. Then, ethanol was replaced by a solution composed of 40% bleach in distilled water containing three drops of tween 80 (Sigma-Aldrich P4780-500 mL). The seeds were soaked in this solution for 30 min with gentle agitation, and then rinsed at least four times with sterile distilled water. Hot (~50°C) autoclave sterilized half strength Murashige and Skoog (MS/2) medium (250 mL) is poured in Petri dishes, and let solidify for ~45 min. The MS/2 solid medium is composed of 2.15 g.L-1 of MS medium basal salt mixture (Duchefa Biochemie, M0221), 75 mg.L-1 of MS vitamin mixture (Duchefa Biochemie, M0409), and 8 g.L-1 of agarose type II (Sigma-Aldrich, A6877). Sterile seeds are then pushed into the solidified MS/2 medium with the radicle oriented downwards. Roots were harvested after 6 days of growth.

#### Four-week-old plants

After 3 days of germination in water, the seedlings were transferred to a hydroponic system under controlled conditions (day/night rhythm: 12/12 h, 28/25°C, light intensity: 500 μE m-2 s-1, relative humidity: 55%). Hydroponic system consists of a 50 L plastic box containing 30 L of hydroponic solution on which a 1 cm thick foam mattress is laid. The foam mattress is pierced with gaps to maintain seedling stem bases. The hydroponic medium is composed of (NH_4_)_2_SO_4_ (0.5 mM), MgSO_4_.7H_2_O (1.6 mM), Ca(NO_3_)_2_.4H_2_O (1.2 mM), KNO_3_ (0.7 mM), FeSO_4_ (0.1 mM), Na_2_EDTA (0.1 mM), MnSO_4_. H_2_O (1.7 μM), (NH_4_)_6_Mo_7_O_24_.4H_2_O (0.2 μM), ZnSO_4_.7H_2_O (0.2 μM), CuSO_4_.5H_2_O (0.2 μM), H_3_BO_3_ (1.4 μM), and KH_2_PO_4_ (0.6 mM). The solution is aerated by a pump placed at the bottom of the plastic box and changed every 10 days. The pH was adjusted and maintained at 5.4 ± 0.2.

#### Fresh root sectioning

The tips of growing radicles or lateral roots (2 cm) were cut using a sharp blade and placed parallel to each other to align the root tips (Supplementary Figures 1A,B). They were embedded in one drop of 3% melted agarose (50°C; Supplementary Figure 1C). Patches containing root tips were inserted into a 3 × 1 × 1-cm well filled with 3% melted agarose (Supplementary Figure 1D). After solidification, blocks were resized and glued on a vibratome plate to be sliced. The vibratome (Hm650v (Thermo Scientific Microm)) parameters were speed 30, frequency 70, amplitude 0.8, and thickness 60 μm (Supplementary Figure 1E). Sections were transferred either onto chamber slides (Lab-teak 177402) for immunostaining (Supplementary Figure 1F), or onto slides humidified with 1X alkaline phosphate buffer (PBS, Sigma-Aldrich P3813) for observation.

#### Immunolocalization

The sections placed on the chamber slides (three per chamber) were first rinsed in 0.1 M glycine supplemented with 1X PBS and then twice in 1X PBS, each for 10 min. The tissues were then immersed in a PBS solution containing 5% bovine fetal serum (blocking solution, Thermo Fisher 37520) at 4°C overnight under agitation. Primary antibodies, diluted 1/10 in this blocking solution, were applied overnight at 4°C under agitation. The sections were then rinsed 3 times in PBS 1X for 10 min. The root sections were incubated for 2 h with the secondary antibody diluted 500-fold in blocking solution under agitation. These antibodies were coupled to a fluorophore, Alexa 546 anti-rat antibody (Invitrogen A11081) or Alexa 546 anti-mouse antibody (Invitrogen A11060). Then, the sections were rinsed again three times in 1X PBS under agitation, for 10 min each. The chambers were removed and few drops of mowiol mounting media (Sigma-Aldrich 81381) were added. A coverslip was placed on the root sections, which were allowed to dry for 36 h at 4°C in the dark.

#### Paraplast fixation

Radicles grown on Petri dishes for 6 days were fixed in 4% paraformaldehyde (in PBS 1X) overnight at 4°C and rinsed twice using PBS 1X (Jackson, 1991). The fixed tissues were dehydrated in ethanol, cleared in Histochoice Clearing Agent (HistoClear, Sigma Aldrich), and embedded in Paraplast (Fisher). The tissues were sectioned (6 μm thick) on a Leica RM2255 microtome and mounted on SuperfrostPlus slides (Fisher).

#### Microscopy

Bright field and autofluorescence observations were performed using a Leica DM4500 microscope. For autofluorescence, images were taken with the “A” filter cube (excitation range: UV; excitation filter: BP 340–380; suppression filter: LP 425). Immunostained sections were observed with confocal microscopes: Zeiss LSM 510 or Leica SP8. The cell walls were first visualized using autofluorescence [720-nm (chameleon laser) or 405-nm respectively]. The secondary antibody was visualized using a Helium/Neon laser at 543 or 561 nm respectively. Pictures were taken with a color Retiga 2000R camera (QIMAGING, Canada) running Volocity image acquisition software (Improvision, UK).

## Author contributions

SH, FD, MB, CB: acquisition of data; SH, CP, AD analysis of data; SH, EG, CP, AD: drafting of the manuscript.

### Conflict of interest statement

The authors declare that the research was conducted in the absence of any commercial or financial relationships that could be construed as a potential conflict of interest.

## Abbreviations

ae: aerenchyma
c: cortex
ed: endodermis
ep: epidermis
ex: exodermis
gt: ground tissue
ic: inner cortex
L-LLR: Large-type Large Lateral Root
oc: outer cortex
sc: sclerenchyma
SLR: Small Lateral Root
st: stele
T-LLR: Thin-type Large Lateral Root
x: xylem.
