# Supplementary material for: Immunoprofiling of Rice Root Cortex Reveals Two Cortical Subdomains
Source: Front Plant Sci. 2016 Jan 7;6:1139. doi: 10.3389/fpls.2015.01139 (PMC4703777; doi:10.3389/fpls.2015.01139)
Supplement: Supplementary file 1 [file Image1.pdf]

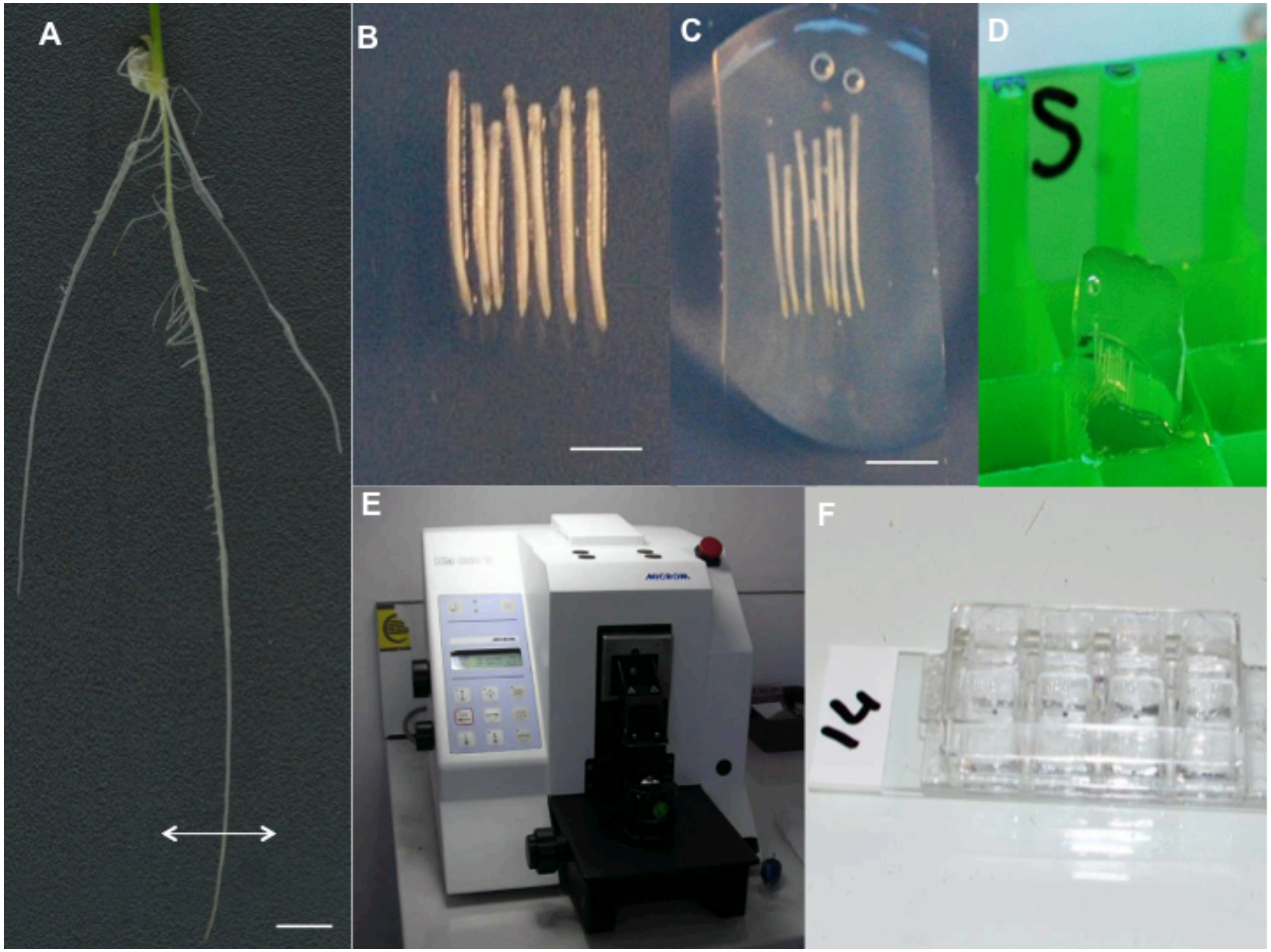

Suppl-Figure1. Medium throughput protocol to produce radial sections of rice radicle meristems. A. Six-day-old rice seedlings grown on MS/2 medium. Radicle root tips (2 cm long) were collected using a sharp blade (arrow). **B.** Samples were placed parallel to one another to align the root tips. **C**. The root tips were embedded in one drop of 3% melted agarose (50°C). Bar= 0.5 cm. **D**. A patch containing root tips was inserted in a 3x1x1-cm well filled with 3% melted agarose. **E.** After solidification, the blocks were resized and glued on a vibratome plate to be sliced. **F.** The roots were sectioned (60 µm thick) radially ≈1 cm above the root tip, and the sections were placed on chamber slides for immunostaining (three sections per chamber). Bar= 0.5 cm.
